# Minimally Invasive Subacute to Chronic Subdural Hematoma Evacuation with Angled Matchstick Drill and Repurposed Antibiotic Ventriculostomy Catheter Augmented with Alteplase: A Technical Case Report

**DOI:** 10.7759/cureus.6049

**Published:** 2019-11-01

**Authors:** James Dorosh, Marcus F Keep

**Affiliations:** 1 Neurosurgery, Sanford Medical Center, Fargo, USA; 2 Neurosurgery, Sanford Brain & Spine Institute, Fargo, USA

**Keywords:** minimally invasive neurosurgery, chronic subdural hematoma evacuation, subacute subdural hematoma evacuation, alteplase augmentation, ventriculostomy catheter, subdural hematoma

## Abstract

The operative management of subacute subdural hematomas (sSDHs) and chronic subdural hematomas (cSDHs) in the elderly is complicated by age itself, multiple medical comorbidities, and anticoagulant and antiplatelet medications; therefore, the search for less invasive, yet more effective, treatment techniques has become a goal. Here, we present the use of a repurposed ventriculostomy catheter in the minimally invasive drainage of a mixed sSDH with the residual solid clot component subsequently liquefied with local alteplase (tPA) administration in an elderly female producing effective hematoma and symptom resolution.

## Introduction

Chronic and subacute subdural hematomas are a common malady faced by the neurosurgeon. Most commonly, SDHs arise from the tearing of bridging veins as they transverse the space between the arachnoid and dura mater, though, in a significant minority of cases, an arterial source is responsible [1-2]. SDH outcome and prognosis are highly variable and depend on several factors, including patient age, presenting Glasgow Coma Scale (GCS) score, premorbid Modified Rankin Scale, smoking, fever, and the use of anticoagulation and antiplatelet medications [1,3].

Overall, the chronic subdural hematoma (cSDH) incident rate has been steadily increasing as the population ages, with as many as 20.6 per 100,000 persons affected [1]. The peak age of onset has increased too, with patients in their 70s now representing the largest demographic of cases in some populations [4]. Because these elderly patients frequently possess complicated medical histories and multiple comorbidities, finding even more minimally invasive treatment techniques than the traditional double burr hole washout technique is desirable.

Patients are stratified into those requiring surgery and those to be treated conservatively. Patients with a rapidly deteriorating neurological condition, unilateral or bilateral dilating pupils, or posturing require emergent evacuation. Symptomatic patients with subacute and chronic subdural hematomas of 10 mm depth and those with more than a 5 mm midline shift are deemed operative [5].

Amongst those requiring an operation, burr hole surgery - placing two large burr holes of standard 12 mm diameter using a craniotome with washout of the subdural space between them, often with a red rubber catheter - is frequently performed as first-line therapy. The double burr hole technique is favored as less invasive with decreased morbidity and complications when compared to full craniotomy, which opens a larger window through the skull [3,6]. The additional placement of a Jackson-Pratt style drain for up to several days in conjunction with double burr hole drainage has been shown to decrease SDH recurrence rates and improve six-month mortality in a randomized control trial [7]. Each of the two burr holes typically requires two 3 cm incisions, and, for at least the anterior frontal burr hole, to be covered over with a round titanium miniplate to prevent leaving a visible and palpable indentation in the more prominent anterior skull and scalp. Age-related alopecia and androgen pattern balding often make these scalp indentations and scars easily visible and identifiable as the result of a neurosurgical operation.

Another effective approach to cSDH management is the minimally invasive subdural evacuating port system (SEPS) [8-9]. This system uses a narrow gauge threaded hollow metal conductor tube that is placed 90° perpendicular to the skull surface. Through a short skin incision of less than 1 cm, the conductor penetrates the skull and dura via twist drilling to drain the hematoma with negative pressure created by connecting the silicon tubing with grenade bulb suction. With SEPS, no tubing enters the subdural space, which prevents it from perforating the brain despite the use of its direct 90° angle trajectory from the surface of the skull [9-10]. The SEPS system possesses several advantages with regards to cosmesis. Because the skin incision and twist drill hole are small, no covering plate is necessary and patients are left with little scarring and no visible skin indentation. If the two burr hole or SEPS technique does not sufficiently evacuate the cSDH, the follow-on surgery is usually more invasive craniotomy. Craniotomy gives visualization and physical access to remove the cSDH inner and outer membranes in affected patients, allowing the brain to re-expand to fill the subdural space [11].

The Camino bolt system - designed for intracranial pressure monitoring and accessing the ventricular system - has also been used to drain cSDHs. The Camino bolt uses twist drill perforation and hollow screw fixation through the skull via a short skin incision with minimal skull perforation. Per a case report, it has been shown to drain a cSDH with successful augmentation using alteplase [12].

Here, we present our minimally invasive technique. We use a repurposed antibiotic ventriculostomy catheter with gentle gravity siphon drainage over three days. The catheter is placed into the subdural space through a single narrow-gauge 45° angled cranial perforation made with a matchstick burr on a high-speed drill. As needed on a case-by-case basis, residual nonflowing still coagulated subdural blood seen on the first or second postoperative CT scan is broken up and liquefied with alteplase (tPA) infusion for up to three days of drainage. Here, we describe the management of a subacute subdural hematoma (sSDH) in the case of a 92-year-old female.

## Technical report

A 92-year-old female with an extensive medical history and multiple comorbidities on 325 mg of daily aspirin presented to the emergency department, transferred from an outside facility, with difficulty standing, dysarthria, and slowed cognition worsening over the past week. She reported falling from a standing height 16 days ago, striking the right side of her head and right hip. She had one week of difficulty rising to the standing position during which she developed slowed cognition and garbled speech. Retrospectively, her neurological function on the Markwalder's grading scale (MGS) was at the middle point (2) on the 0 to 4 range [13-14].

**Figure 1:**
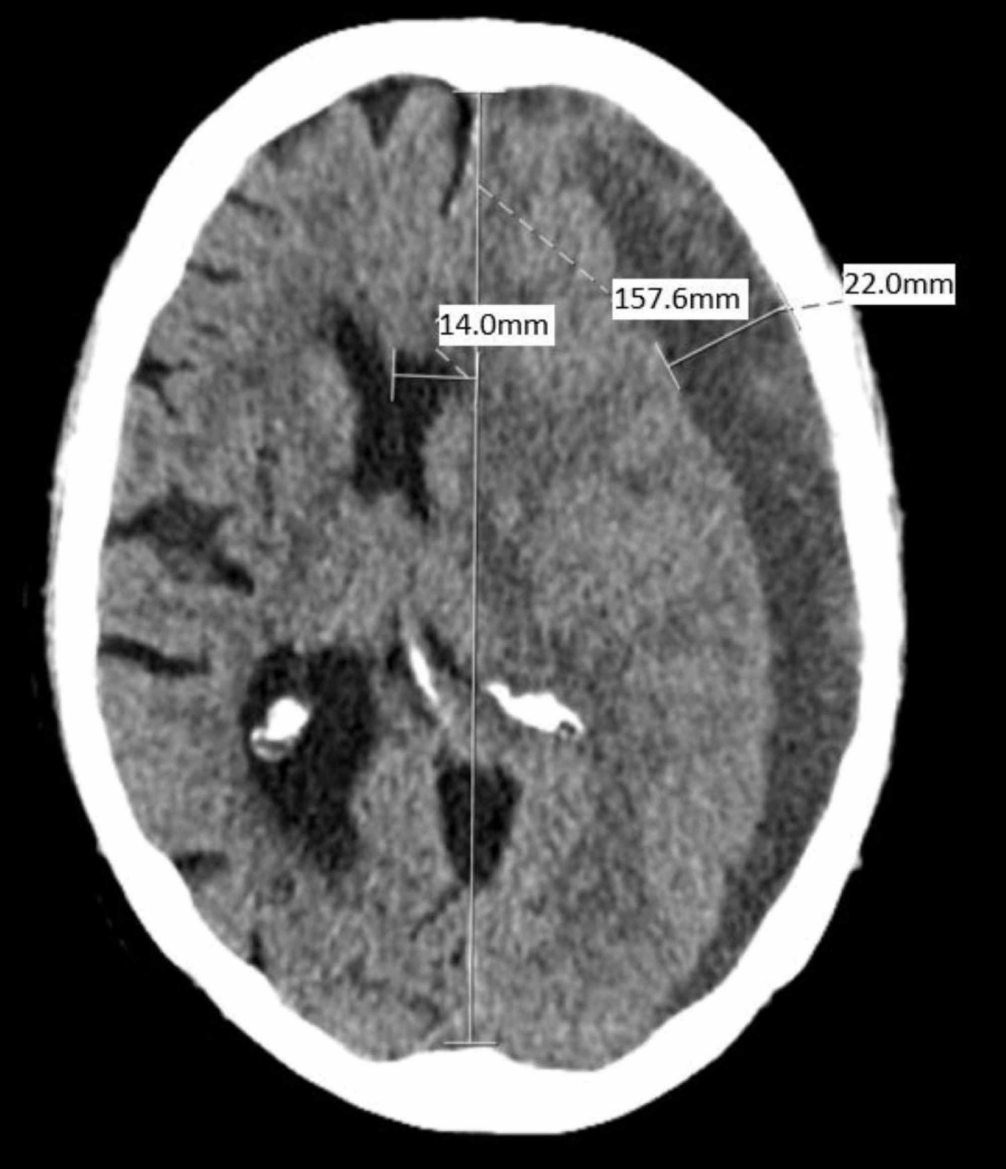
The patient’s presenting CT examination demonstrating a large, left-sided, crescent-shaped frontoparietal sSDH measuring 22 mm at the deepest point, with 14 mm of midline shift, left to right. The SDH has a mixed density, with components varying from hypodense to isodense compared to the brain. CT: computed tomography; sSDH: subacute subdural hematoma

The CT scan demonstrated a 22 mm deep, acute on subacute, mostly hypodense SDH overlying the left panhemisphere, producing a 14 mm left to right shift (Figure 1).

Within the central aspect of the sSDH, there was a 1 cm area of hyperdensity likely corresponding to a recently bleeding bridging vein. On physical examination, the patient had slowed cognition, demonstrated 5/5 motor strength throughout, was weaker on the right side, and had 2+/4 right-sided reflexes of the biceps, triceps, patellar, and Achilles. She also had a bilaterally upgoing Babinski sign, stronger on the right than the left. She was transfused with platelets and given levetiracetam 1000 mg for seizure prophylaxis in the emergency department.

The patient was taken to the operating theatre for sSDH evacuation. She was placed supine on the operating table, and general anesthesia was induced with endotracheal intubation. The anesthesiologist's preference was for general anesthesia, to protect the patient's airway in the lateral position, even with this minimally invasive procedure. Since this patient's case, in more high-risk medical patients, we have used monitored anesthesia care (MAC) sedation and the injection of a scalp local anesthetic. She was positioned in near lateral orientation, right side down, with large shoulder role and soft doughnut silicon rubber padding under the right head for the immobilization and maintenance of neutral cervical alignment, giving access to the left parietal scalp and ease of frontal 45° angle trajectory. The site of maximal sSDH thickness was determined using external head landmarks correlated with the CT scan seen in three orientations, and the overlying hair was clipped. The distance from the planned cranial perforation to the anterior aspect of the sSDH was measured on the CT sagittal plane view for determining the planned length of the to be inserted catheter. The patient was prepped with iodine scrub and draped in the usual sterile fashion. Two grams of Ancef were administered prior to incision. Bupivacaine 0.5% with epinephrine was injected into the scalp at the planned incision site to assist with hemostasis. A scalpel with a 15 blade was used to create a 1-cm skin incision down to the skull. Bipolar cautery was used to achieve hemostasis. The Heiss mini-retractor gave visualization of the bone. A matchstick burr was employed to create a 0.5 cm diameter tunnel angled 45° through the skull surface directed anteriorly towards the patient’s forehead, taking care to not prematurely perforate the dura before it was coagulated. The underlying dura was visualized and a small curette was used to remove the residual bone. The dura was cauterized with monopolar coagulation with an insulated shaft. The cauterized avascular dura was penetrated with the active monopolar tip, which was removed simultaneously with an ejection of dark brown blood under moderate pressure. The antibiotic ventriculostomy catheter was inserted and directed toward the forehead through the 45° cranial perforation to a depth of 3 cm from the outer skin. The stylet was removed to prevent entry into the brain and the soft catheter was fed into the subdural space with bayonet forceps for a total length of the premeasured 10 cm. The distal end of the catheter was tunneled under the skin to an exit stab point 1 cm posterior to the incision along the straight path of the drill hole. A small piece of gelfoam was placed into the burr hole around the catheter to reduce air and acute blood from the skin edge entry into the subdural space.

The incision was irrigated with antibiotic saline and 3% hydrogen peroxide prior to closure with interrupted 3-O vicryl followed by skin staples. The catheter was affixed to the skin with three individual 3-O vicryl sutures using the Roman sandal technique to reduce the chance of early unintended catheter removal. The incision and drain site were covered with mupirocin antibiotic ointment and Telfa bandage was affixed to the scalp with staples. The distal tubing of the catheter was attached in sequence to the luer nipple secured with a silk tie, the T-connector for later tPA administration, two IV extension tubings to give adequate length for over-the-side-of-the-bed gravity siphon drainage, a double male connector, and, ultimately, an external ventriculostomy drainage (EVD) bag. The EVD bag was placed over the side of the patient’s bed, 6 inches off the floor, using a fluid column gravity siphon for gentle continuous negative pressure. Fifty ml of dark brown, low-viscosity fluid drained into the bag in the OR.

**Figure 2:**
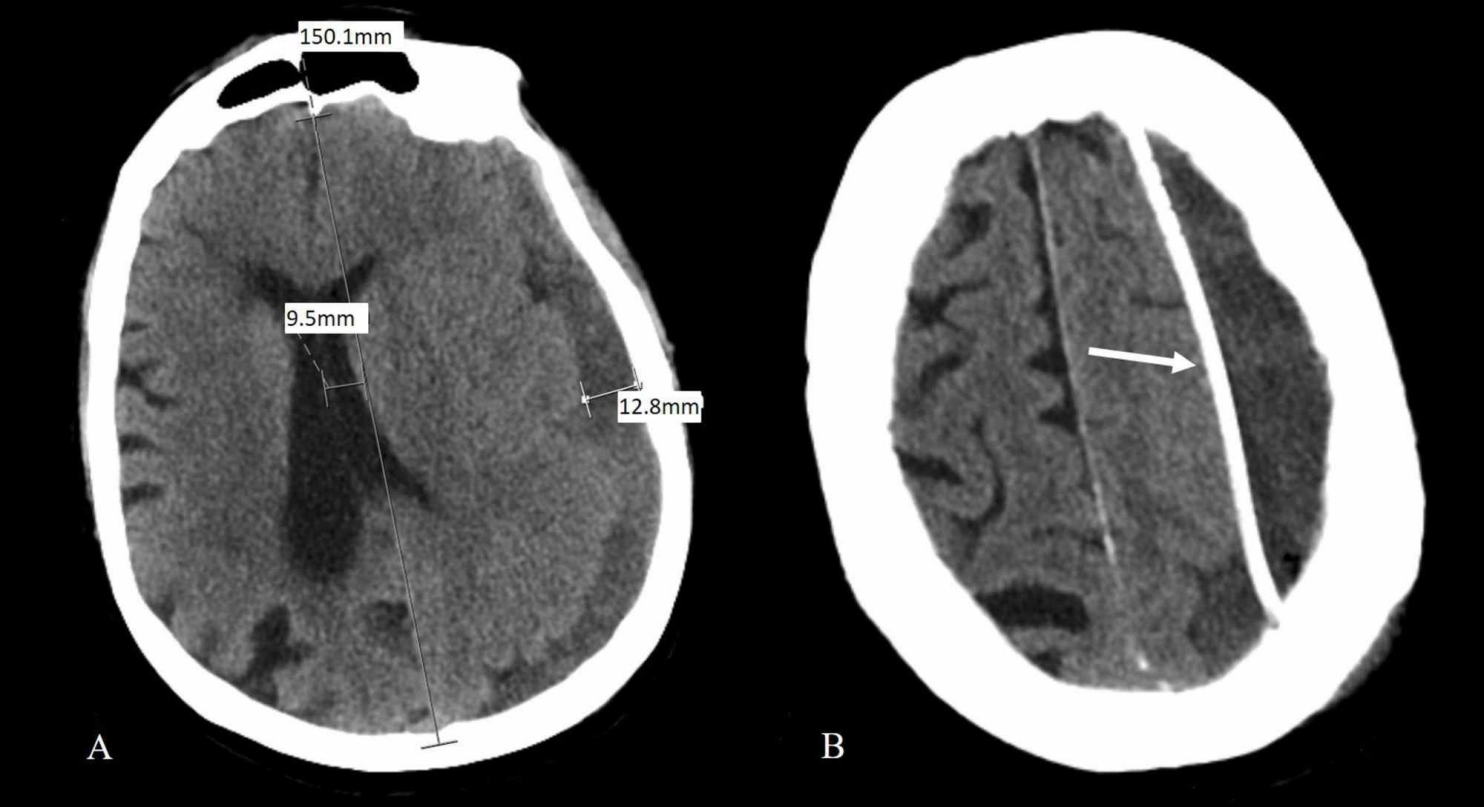
POD 1 CT examination shows the midline shift improved to 9.5 mm with a maximal hematomal depth of 12.8 mm. Residual SDH is relatively more isodense with the brain, suggesting blood is more recent and potentially still coagulated (A). Also note interval catheter placement to the superior aspect of hematoma (B). POD: postoperative day; CT: computed tomography; SDH: subdural hematoma

Postoperative day (POD) 1: 120 cc of dark-colored blood was drained since catheter placement. Drain tubing was noted to be patent. The first postoperative CT scan head demonstrated an improvement of left to right shift from 14 mm to 9.5 mm (Figure 2a) with appropriate catheter placement on the superior aspect of the persistent residual large remaining hematoma (Figure 2b). The patient reported considerable symptom improvement.

POD 2: The patient’s drainage was minimal, and the second postoperative CT imaging showed the still substantial remaining SDH. A decision was made to administer alteplase into the subdural space to break up the coagulum. Using sterile gloves and iodine swab cleansing of the T-valve entry port, the Off valve was set toward distal flow - creating a fluid path between the entry port and the subdural space. A single dose of 2 mg of alteplase tPA in 2 cc of preservative-free saline was infused through the proximal catheter into the subdural space followed by infusion of 10 cc of preservative-free saline, to allow wide dispersal of the tPA throughout the subdural space. The catheter T-valve was kept in the off position distally for one hour. Thereafter, the valve off was set toward the sealed port, re-establishing the fluid pathway from the subdural space to the EVD bag. The catheter resumed the same gravity siphon drainage.

**Figure 3:**
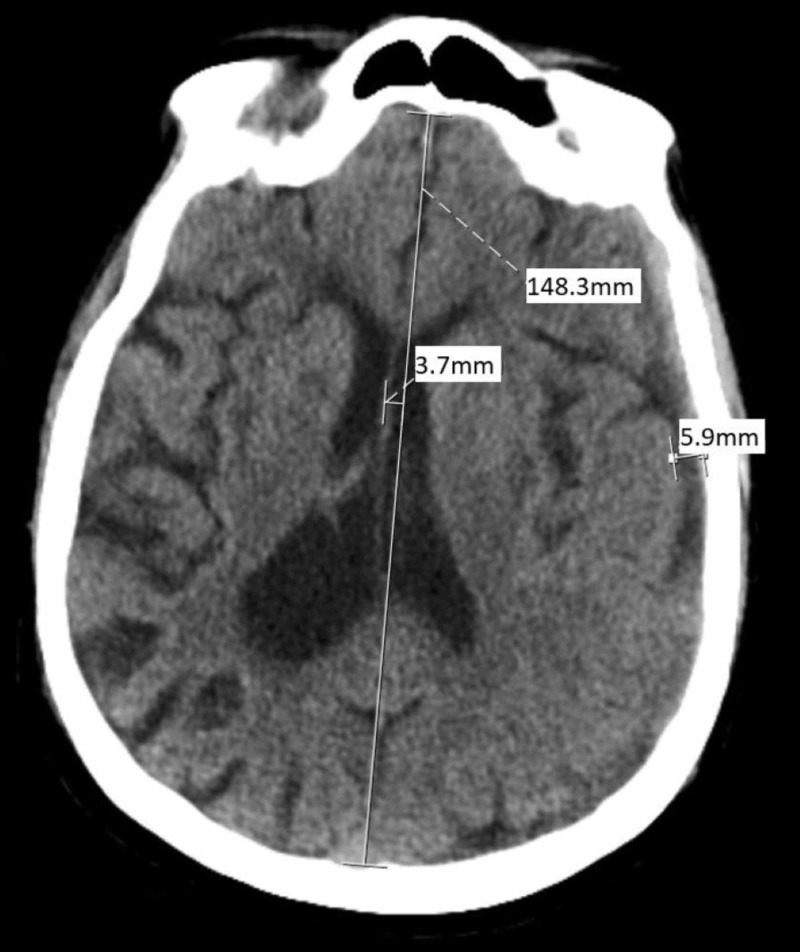
POD 3 CT examination completed 11 hours after 2 mg infusion of tPA demonstrates continued hematomal drainage with only 3.7 mm left to right midline shift and maximal SDH depth reduced to 5.9 mm. POD: postoperative day; CT: computed tomography; tPA: alteplase; SDH: subdural hematoma

POD 3: 100 cc of dark red blood was drained, and CT imaging showed improvement of the SDH size, which had decreased to a depth of 5.9 mm, with a further decreased left to right midline shift from 9.5 mm down to 3.7 mm (Figure 3). The patient reported complete symptom alleviation. The evacuation was determined to be adequate and there was no need for a second alteplase infusion. The drainage catheter was removed and the skin stapled closed.

**Figure 4:**
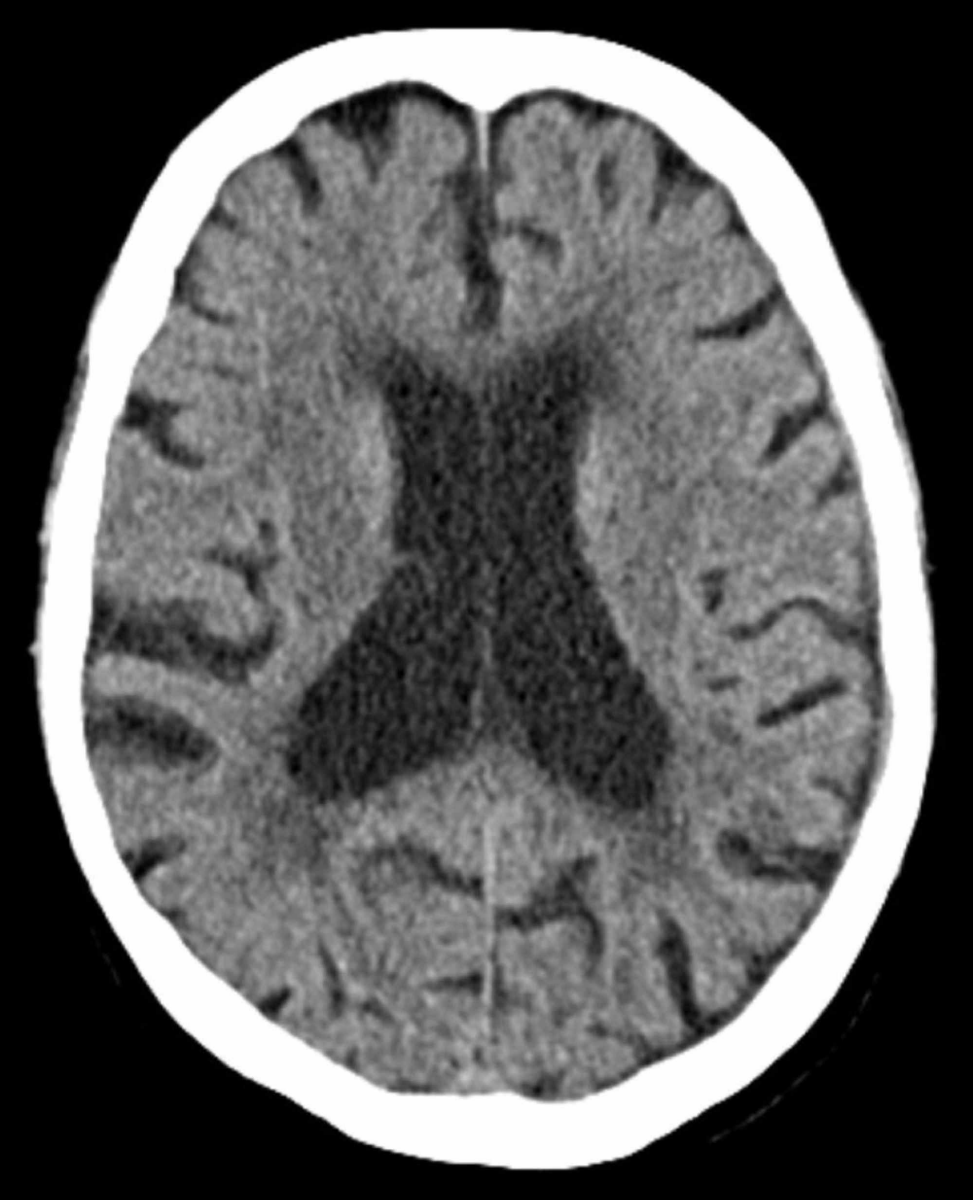
Ten-week outpatient follow-up CT examination demonstrating persistent thin subdural collection with minimal mass effect and no significant midline shift. CT: computed tomography

The patient was monitored closely without symptom recurrence, rebleeding, or other complications and discharged on POD 6 for a total length of stay of six days. Levetiracetam was discontinued at four weeks. At her four-week and 10-week follow-up appointments, the patient continued to deny any symptom return. On physical exam, her strength was 5/5 bilaterally and her CT imaging at 10 weeks demonstrated minimal remaining SDH with no significant midline shift (Figure 4).

## Discussion

The procedure described here offers several advantages. This approach is less invasive than double 12-mm burr hole surgery and craniotomy since only a single small 5-mm diameter cranial perforation is created. This decreases the total time under general anesthesia and can be completed under local anesthesia alone. Because the entry perforation diameter is small, there is improved cosmesis, and patients are less likely to note scalp indentation. As the placement of a drain is now recommended during the primary drainage of cSDHs, this procedure ensures drain usage during all applications.

Our case also demonstrates the augmentation of catheter-based sSDH drainage with alteplase (tPA) administration in the presence of a partially coagulated hematoma. The addition of tPA is a useful technique in the drainage of subacute and cSDHs, especially in those patients that are poor surgical candidates or refuse reoperation [15]. We chose a dose of 2 mg of alteplase since this is the dose used to clear central line catheters, and we wished to have no more than two alteplase injections 24 hours apart. The catheter was clamped for one hour to give sufficient time for the alteplase to interact with the clot. We kept the possibility to repeat the injection of 2 mg alteplase once in 24 hours after a CT scan to confirm no new bleeding. The one-day interval was chosen to give time for the maximal drainage of the SDH before assessing if a repeat dose was needed. Ten cc of preservative-free saline flush was chosen because of the relatively large potential subdural space that was already partially decompressed by the initial drainage and to break up the clot at a distance from the catheter tip.

Two mg alteplase is the dose used to open central venous catheters obstructed by a blood clot and is repeatable once after 120 minutes [16]. The minimally invasive surgery plus alteplase for intracerebral hemorrhage evacuation (MISTIE) trial used a brain catheter to deliver alteplase for intraparenchymal cerebral hemorrhage breakup and drainage at a finalized dose of 1 mg with 3 cc of preservative-free normal saline flush and one-hour clamping before release to the drain. CT scans were performed between each dose, and alteplase dosing was allowed every eight hours up to nine total doses or until the hematoma was evacuated [17]. The Camino bolt SDH evacuation augmented with tPA case report used alteplase 0.5 mg with clamping for 30 minutes [14].

The addition of tPA should be considered carefully in those with concern for hemostasis, contusion, or cerebral hemorrhage. The worrisome complication of subdural tPA would be localized bleeding, usually in the form of an acute SDH [15]. We have not experienced this problem in over 25 cases of alteplase-augmented SDH evacuation at Sanford Medical Center, Fargo.

There are several other potential complications that can occur during the drainage of a subacute and cSDH via a catheter-based approach or with the placement of any drain into the subdural space. One must avoid direct trauma to the brain surface, which can cause parenchymal injury and hemorrhage. As described above, the risk of this complication can be reduced with the use of an angled catheter approach approximately 45° below the parallel of the skull’s surface. This angulation helps prevent the catheter from damaging the brain surface. Further, removing the stylet by the 2 cm or, at the most, the 3 cm mark from the skin surface makes the rigid catheter supple and less likely to penetrate into the brain parenchyma. Not removing the stylet will result in the catheter entering the brain parenchyma. The selection of patients with thicker SDHs provides a physical buffer from parenchymal injury. To benefit from the presence of the thick subdural buffer, the catheter should be placed immediately before more than minimal subdural blood has drained. This also limits the amount of air able to enter the subdural space to reduce postoperative pneumocephalus. If rebleeding were to occur, it is appropriate to progress to craniotomy. Empyema and infection of the subdural space have high morbidity and mortality. Efforts to reduce the chance of infection include peri-operative antibiotics, the use of an antibiotic-impregnated catheter, complete evacuation of the subdural hematoma, and catheter removal no later than the third postoperative day.

## Conclusions

A single case and method of treatment is presented here: the use of a repurposed antibiotic ventriculostomy catheter with a closed gravity three-day drainage system through a single 5-mm diameter 45° angled cranial perforation via a 1-cm skin incision is a low invasive approach to subacute and cSDH management. In some cases, despite adequate drain placement, there is persistent residual, more acute nonliquid blood coagulum in the subdural space compressing the brain; this can increase the risk of another SDH and potentially require more invasive follow-on surgery. Alteplase is effective to liquefy that residual coagulum to give complete SDH evacuation via a subdural catheter without further operation.

## Appendices

Materials used

1a) Medtronic - ARES Antibiotic Impregnated Ventricular Catheter 23 cm. Ref 91101

2) Integra - Polycarbonate F8 Luer Lock Connector. Ref 999108

3) Smiths Medical - Hi-Fl™ 3-Way Stopcock. Ref MX4311L

4) B.Braun - Extension Set with Female Luer Lock Connector & Spin-Lock® Connector. Ref 473012

5) Smiths Medical - Double Male Luer Lock. Ref MX493

6) Medtronic - Duet External Drainage Bag. Ref 46912

7) Antibiotic saline: Bacitracin 50,000 units and gentamicin 80 mg per liter of normal saline
